# Oral Spermine Supplementation in Gestated Rabbit: A Study on Villi Height of Immature Intestines

**DOI:** 10.3389/fsurg.2021.721560

**Published:** 2021-09-09

**Authors:** Riana Pauline Tamba, Yefta Moenadjat

**Affiliations:** Faculty of Medicine, Department of Surgery Cipto Mangunkusumo General Hospital, Universitas Indonesia, Jakarta, Indonesia

**Keywords:** spermine, rabbit, gestation, immature intestines, villi height

## Abstract

**Introduction:** Immature intestines are the major problem in prematurity. Postnatal oral spermine has been shown in studies to improve intestinal maturation in rats and piglets. This study aimed to find out the efficacy of spermine in rabbits during gestation.

**Method:** An experimental study was done in an unblinded, randomized manner on those treated with and without spermine administration. A morphological examination of hematoxylin–eosin-stained villi was performed under a light microscope with a focus on villi height. Data were subjected to analysis.

**Results:** The median of the spermine-treated group was found to be higher at 24, 26, and 28 days than the non-spermine group, but was not significantly different.

**Conclusion:** Oral spermine supplementation during gestation might improve intestinal villi height in immature rabbit intestines.

## Introduction

Premature birth was associated with a high risk of developing gut-derived infection and intestinal immaturity, as well as a 50% risk of death (1). According to one study, 90% of neonatal deaths were caused by gut–derived infections associated with intestinal immaturity (1). Thus, intestinal immaturity is a major problem in premature infants (2). In the term neonate, intestinal maturity is found in the first 3 weeks of life. However, premature babies' maturity takes longer, although there is no clear definition. The various anatomical and physiological structures of the cells/tissues constructing the digestive tract are not fully developed yet in immaturity. The epithelium of fetuses has not yet developed into the adults' type of epithelium, which is found in four types: enterocytes, entero(neuro)-endocrine, goblet cells, and Paneth cells, and no mucosal integrity assembling the epithelial lining. Likewise, microfold cells (M cells) and Peyer patches, lymphoid tissue in the lamina propria as gut-associated lymphoid tissue, play a role in inducing the defense system, in addition to the insufficient mucus layer (3, 4). Lamina muscularis, which plays a role in propulsion, is also found to be insufficient. Oral intake, including breastfeeding, is frequently delayed in this condition because it may be followed by severe effects associated with propulsion (3). On the other hand, the homeostasis of commensal bacteria, which is influenced by oral intake, has not been achieved. In contrast, commensal bacteria are known to induce maturity and gastrointestinal mucosa's natural defense system as well (4–7).

In addition, mucin insufficiency, and mucosal permeability that is not achieved due to unassembled integrity of epithelial lining, allow bacterial translocation. Thus, immaturity of the gastrointestinal leads to sepsis, which is fatal (3). Besides sepsis, the clinical entity associated with intestinal immaturity in preterm neonates is necrotizing enterocolitis (NEC), which is also fatal (8, 9). The immature intestine reacts to molecular patterns of colonizing bacteria and endogenous inflammatory stimuli by mounting excessive inflammation, a hallmark of NEC, due to developmental immaturity in the innate immune response gene (3).

Studies focused on gastrointestinal immaturity have shown a positive effect of oral spermine administration followed by intestinal maturation in mice (10–13) and piglets (14, 15). Polyamines (spermine, spermidine) are ubiquitous low-molecular-weight polycationic compounds that play an essential role in cell proliferation, growth, and differentiation in various cells/tissues (16, 17). In the digestive tract, spermine is known to interact with the constituent proteins of the intestinal barrier and play an essential role in wound healing and the immune system (13, 15, 18). The role of spermine was the interplay of molecules resembling epithelial junctions of the intestinal mucosa and associated cytoskeleton molecules responsible for providing an intestinal barrier. These studies showed achievement of intestinal maturity following postnatal spermine administration with different parameters. The morphological parameters investigated were villi height and crypt depth (19). Another study focused on biochemically showing the permeability and expression of junctional proteins, cytokines, etc (20, 21). However, spermine administration's efficacy during gestation is yet unknown. Therefore, this study focused on spermine administration during gestation to determine the efficacy of postnatal maturation in premature newborns, focused on the height of intestinal villi. It was hypothesized that the maturity level of newborns soon after delivery is achieved, but not a mature level in the first 3 weeks of a newborn's life.

## Materials and Methods

An experimental study was carried out on 3 kg weighted New Zealand White adult female rabbits (*Oryctolagus cuniculus*) prepared for a study by The Animal Lab, Ciawi, Bogor. Twenty–four rabbits were enrolled in the study. They were fed with standard DM20 pellets, bred, and handled with care during gestation. These rabbits were assigned in an unblinded, randomized manner to those treated with spermine administration and non–spermine administration. The subjects were randomly selected and set in the treatment and control groups, respectively. Spermine of 20 mg per kg body weight once daily was supplemented with food during the gestation period. The dose was set based on a previous study by Peulen et al., converted to a rabbit dose using a human equivalent dose conversion (22). In this study, the crew fed one rabbit and waited for it to finish its food before moving on to the next rabbit to ensure all rabbits ate the whole portion of their meal. Feeding was proceeded with close monitoring to assure these animals take the food completely, which was given once daily.

The prematurity was established at various times, namely 24, 26, and 28 days of gestation, which represents the third trimester of pregnancy in humans, 28–38 weeks. These prematurely born fetuses were enrolled in a parallel assigned, non–masking randomized manner. The gestation was terminated by Cesarean section. The Cesarean section was carried out with ketamine of 10–40 mg per kg body weight and xylazine 3–5 mg per kg body weight, intramuscularly. Fifty fetuses of these rabbits were prematurely born. Furthermore, laparotomy was carried out on the newborn under ketamine per kg body weight and 1.5 mg of xylazine per kg body weight. A sample of terminal ileum measuring 5–6 cm was taken as the specimen for the study. Following laparotomy, the newborns were sacrificed according to the regulations in the animal lab. The control group referred to those normally delivered.

The specimens were stained with hematoxylin–eosin and examined under a light microscope (OptiLab Advance, Miconos) at 10 times objective magnification to determine villi height. The height of the villi of a fetus of a 14-days-old fetus was used as the control (23). Data was subjected to analysis using the ANOVA test. This study was approved by the Committee of Ethics, Faculty of Medicine, University of Indonesia with reference number 18-03-0249. The study was registered on ClinicalTrials.gov (No. NCT04004091).

## Results

**Figure 1:**
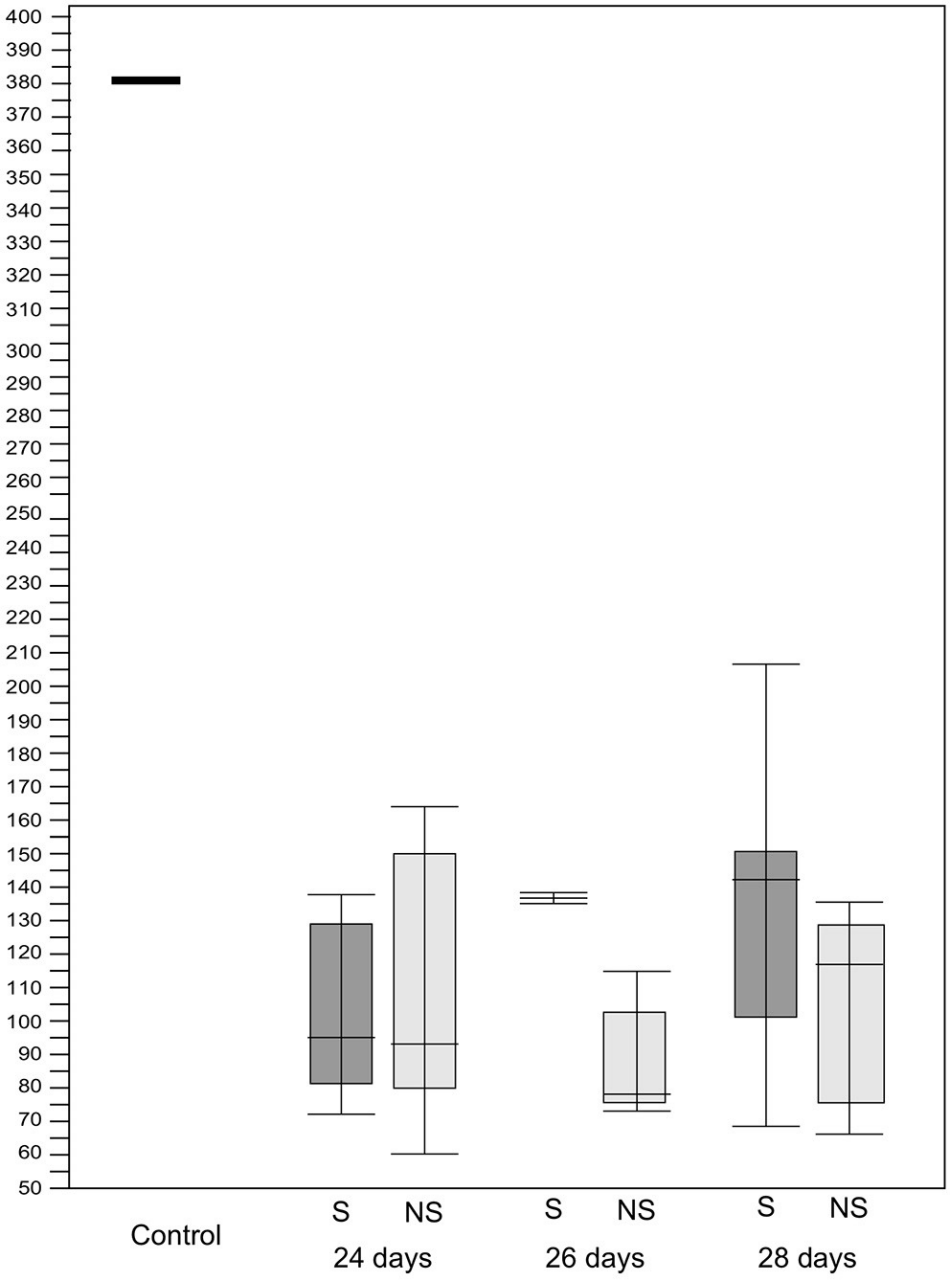
Box–Whisker plot showing the difference in villi height between the spermine–treated and non–spermine groups in the immature intestines. The median of the spermine–treated group was higher than the non–spermine groups in three treated periods, namely 24, 26, and 28 days, though this difference was not statistically significant. For details, see the text.

**Figure 2:**
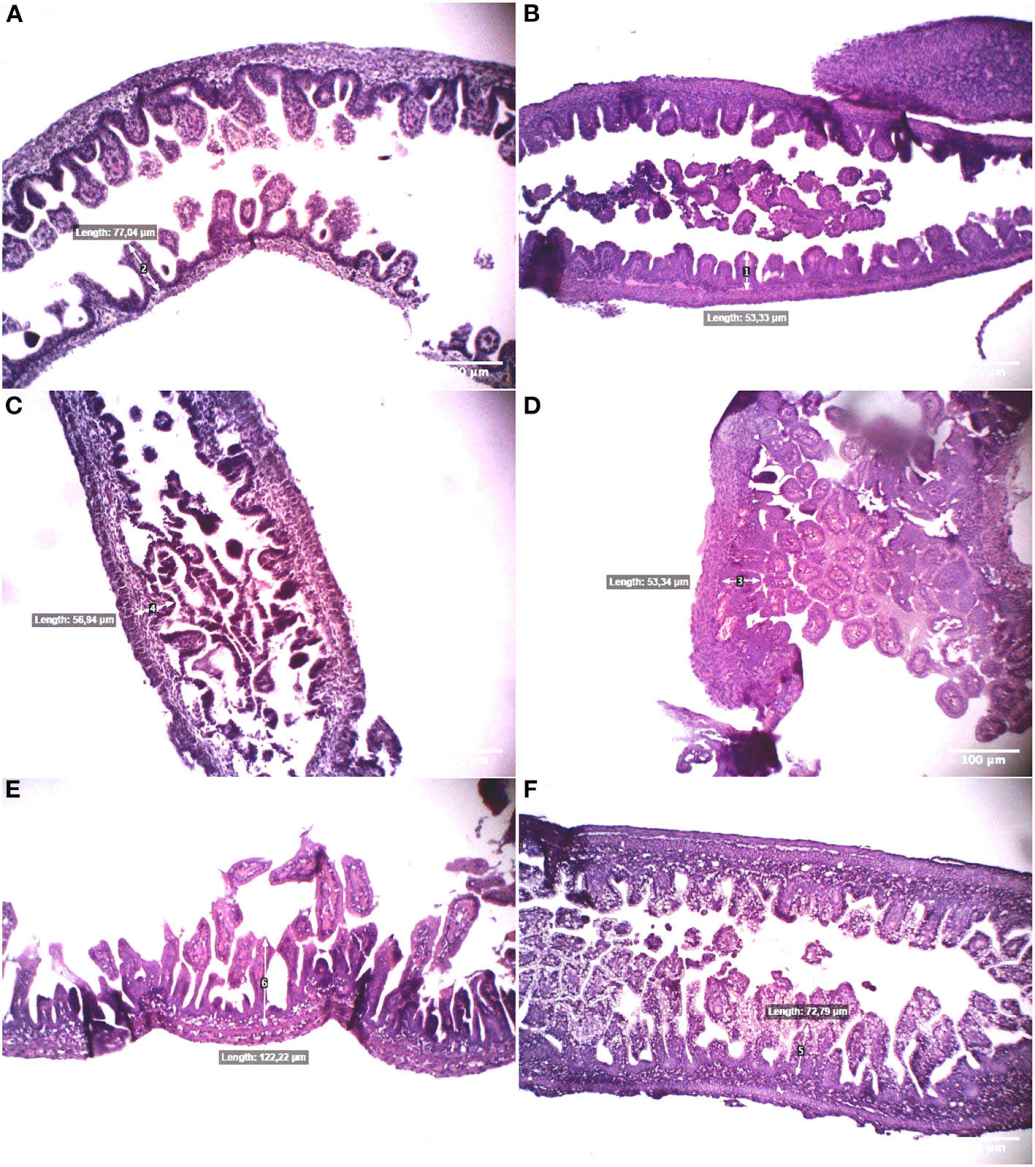
Morphological presentation of villi height in hematoxylin-eosin (HE)-stained specimens of immature rabbit's ileum at 24 days **(A,B)**, 26 days **(C,D)**, and 28 days postnatal **(E,F)** under objective magnification of 10 times. The left-sided figures show those of the spermine-treated group, and the right-sided figures represent the non–spermine group. It is shown that the spermine group's ileal villi are longer than the non–spermine group and more regularly. The spermine group's regular-shaped villi are configured by day 28 postnatal, whereas the non–spermine group remains discretely distributed.

Of the spermine-treated group, seven specimens were 24 days, four specimens were 26 days, and two specimens were 28 days. Of the non–spermine group, four specimens were 24 days, three specimens were 26 days, and four specimens were 28 days. The mean of villi height in 24–, 26–, and 28-days spermine groups was 100 μm + 23.5, 135 μm + 39.142, and 138 μm + 1.0, respectively. While the mean of villi height in non-spermine groups of 24–, 26–, and 28 days were 100.33 μm + 14.88, 102.25 μm + 36.75, and 106.28 μm + 1.0, respectively. The distribution was not the normal one, median (min–max) was used for statistical analysis purposes (Table 1, Figures 1, 2). The difference was not significantly different with *p* values of 24–, 26–, and 28 days were 0.344, 1.000, and 0.355, respectively.

**Table 1:** Ileal villi height in spermine and non–spermine treated groups.

	**Villi height (μm)**	**Spermine (μm) Median (min–max)**	**Non–spermine (μm) Median (min–max)**	***p*-value**
Control	380	–	–	
24 days	–	143 (68–207)	117 (74–135)	0.344
26 days	–	108 (78–115)	95 (72–138)	1.000
28 days	–	138 (137–139)	92.5 (60–164)	0.355

## Discussion

This study is the first investigation focused on the immature ileum of premature rabbits. Unlike the previous studies on mice and piglets, focusing on intestinal maturation after oral spermine administration in the postnatal period, the present study had a different approach. Firstly, this experiment was carried out on rabbits. There were references directly comparing rat and human intestinal epithelial cells, but to date, there are fewer studies carried out on rabbits, particularly those focused on spermine. Although rat intestines' morphology closely resembles humans' intestines, rabbit intestines resemble pH 7.5 as in humans, and the microflora in the intestines is similar to humans (24). For this reason, we use rabbits for study. Overall, rabbits are phylogenetically closer to primates and have a more diverse genetic background than inbred and outbred rodent strains (25). This makes the model a better overall approximation of humans, mimicking human genetic diversity more accurately (26).

Secondly, the treatment, i.e., spermine supplemented food, was given during the gestation period, and the outcome was observed in the intestine of the premature newborn rabbit. Previous studies have shown the efficacy of oral spermine supplementation in intestinal maturation. Maturation is a complex process that may be explained differently, namely, morphologically, and biochemically. Morphologically, the villi height of the crypt represents the parameters observed using a conventional hematoxylin-eosin-stained specimen. However, Goblet cells, Paneth cells, and M cells require a particular staining method. Another aspect is the absorptive parameter, representing the barrier integrity and intestinal permeability using fluorescence and other biochemical properties, such as the expression of some molecules that build the epithelial junctions.

To date, those studies have been carried out on mice and piglets. Because no other parameters exist for rabbits, this study concentrated on villi height and crypt depth as parameters; they are simple, feasible, and reliable for representing maturation. Maturation is the development of cells' individual characteristics through growth, while growth is a physical and quantifiable process in development, which is measurable. Studies have shown that DNA methylation, for instance, shows that maturation represents the development of fetal cells into well–developed (matured) cells (27). Somehow, development is closely related to morphogenesis.

The study by Sabater–Molina et al. showed that the mucosa's polyamine concentration is followed by deepening of the intestinal crypt but is not significantly associated with the mucosa's concentration (14). The study by Peulen et al. empowered the statement (28). Furthermore, Peulen's study showed that the maturation is associated with microflora that synthesized the mucosa's polyamine and uptake by the stem cells in the crypts (29). The regulation, as well as the utilization, is then associated with average growth and development. In rabbits, the crypts can be seen as an invagination of a villi base in the duodenum of a 1-day-old rabbit. Villi, goblet cells, and other glands develop later at the end of gestation. Primitive villi could be seen in the first 21 postnatal days, much higher in 22 days, resembling a cylindric shape in the first 28 days. The villi height increased within days in both the spermine-treated and non-spermine groups, according to the study (30). However, intestinal structure development starts earlier in humans in the first trimester of gestation, and maturation is achieved during delivery (30). The maturation remains, and the maturation remains in process. Thus, insignificancy in the analysis is not a big issue as maturation refers to a dynamic process.

Despite the controversy, most studies have shown the efficacy of oral supplementation, referring to close contact with the mucosa—particularly intestinal villi—associated with an increase in villi height (4, 31), as in this present study. The unanswered question was the influence of spermine supplementation during gestation as the aim of the study. The present study was not designed to determine the evidence regarding polyamines, both maternal and fetal or placental. The reason is that there was sufficient information regarding the ability to cross the placenta barrier (32–34).

In summary, we found that oral supplementation in gestated rabbits showed increased intestinal villi height in the premature rabbit's ileum, achieving a mature newborn's height. Still, the achievement was not mature intestines because, in a mature newborn, intestinal maturation is achieved within the first 3 weeks of life. In other words, Spermine improves intestinal maturity intrauterine—although this finding is not significant.

However, there are limitations to the study. Firstly, the small sample size concerned the 3Rs regulations on using experimental animals. Secondly, the problem of broodstock led to finding samples with appropriate gestation periods and, consequently, different samples in each group. Thirdly, the unanswered question regarding the influence of spermine on intrauterine needs to be elaborated further through an investigation into epithelial molecular junctions.

## Conclusion

During gestation, oral spermine supplementation might improve intestinal maturity of intestinal villi in immature rabbits, as indicated by an increase in height.

## Data Availability Statement

The raw data supporting the conclusions of this article will be made available by the authors, without undue reservation.

## Ethics Statement

The animal study was reviewed and approved by Committee of Ethics, Faculty of Medicine, University of Indonesia.

## Author Contributions

RT designed the study and performed the experiments. YM analyzed the data. RT and YM wrote the manuscript. Both authors contributed to the article and approved the submitted version.

## Conflict of Interest

The authors declare that the research was conducted in the absence of any commercial or financial relationships that could be construed as a potential conflict of interest.

## Publisher's Note

All claims expressed in this article are solely those of the authors and do not necessarily represent those of their affiliated organizations, or those of the publisher, the editors and the reviewers. Any product that may be evaluated in this article, or claim that may be made by its manufacturer, is not guaranteed or endorsed by the publisher.
